# Effectiveness of a Geriatric Assessment-Driven Intervention (GAIN) on Chemotherapy Toxicities in Elderly Cancer Patients

**DOI:** 10.7759/cureus.114399

**Published:** 2026-08-12

**Authors:** Shwetha S, Geetha Jayaprakash, Srujana D, Shakthi K, Stephen Z A, Hemanth S Ghalige

**Affiliations:** 1 Department of Medical Oncology, Employees’ State Insurance Corporation (ESIC) Medical College and Post Graduate Institute of Medical Sciences and Research (PGIMSR), Bengaluru, IND; 2 Department of Pharmacy Practice, Acharya and BM Reddy College of Pharmacy, Bengaluru, IND; 3 Department of General Surgery, Employees’ State Insurance Corporation (ESIC) Medical College and Post Graduate Institute of Medical Sciences and Research (PGIMSR), Bengaluru, IND

**Keywords:** cancer and aging research group (carg) score, chemotherapy toxicity, elderly cancer patients, geriatric assessment, geriatric assessment-driven intervention (gain)

## Abstract

Background: Elderly patients with cancer are highly vulnerable to severe chemotherapy-related toxicities due to age-related physiological decline, multimorbidity, and frailty. Standard performance scales like the Eastern Cooperative Oncology Group (ECOG) are often insufficient for assessing this risk. The Cancer and Aging Research Group (CARG) score is a validated tool for predicting chemotherapy toxicity, while the Geriatric Assessment-Driven Intervention (GAIN) model provides a structured, multidisciplinary framework to mitigate these risks.

Objective: This study aimed to evaluate the effectiveness of the GAIN model in reducing the incidence of severe (grade ≥3) chemotherapy-related toxicities in elderly cancer patients. Secondary objectives included assessing the impact of GAIN on treatment continuity, hospitalization rates, and emergency department visits.

Methods: A prospective, randomized controlled interventional study was conducted over six months in the oncology department of Employees’ State Insurance Corporation (ESIC) Medical College and Post Graduate Institute of Medical Sciences and Research (PGIMSR), a tertiary care hospital in Bengaluru, India. Seventy patients aged 60 years or older with solid tumors initiating chemotherapy were enrolled and allocated to either the GAIN arm (n=35) or the Standard of Care (SOC) arm (n=35). All patients underwent a baseline comprehensive geriatric assessment, and their CARG toxicity risk score was calculated. Patients in the GAIN arm received individualized interventions from a multidisciplinary team (MDT) based on identified vulnerabilities. The SOC arm received routine oncological care. The primary outcome was the incidence of grade ≥3 chemotherapy-related toxicities, graded using National Cancer Institute Common Terminology Criteria for Adverse Events (NCI-CTCAE) v5.0.

Results: The incidence of grade ≥3 chemotherapy-related toxicities was significantly lower in the GAIN arm, with 17 of 35 patients (48.6%) experiencing severe toxicity compared to 26 of 35 patients (74.3%) in the SOC arm (p=0.027). Patients in the GAIN arm also had numerically fewer unplanned hospitalizations (14, 40% vs. 17, 48.6%), emergency department visits (14, 40.0% vs. 16, 45.7%), chemotherapy dose modifications (four, 11.4% vs. eight, 22.9%), and early treatment discontinuations (four, 11.4% vs. eight, 22.9%), though these differences were not statistically significant.

Conclusion: The integration of a GAIN significantly reduces the incidence of severe chemotherapy-related toxicities in elderly cancer patients.

## Introduction

The global population is aging, leading to a significant increase in the proportion of older adults diagnosed with cancer. Individuals aged 65 and older now account for approximately 62.3% (11.3 million cases) of new cancer diagnoses and 71.2% (7.5 million cases) of cancer-related mortality among all cancer cases, as reported in an Indian study [1]. Despite this, older adults remain underrepresented in clinical trials, resulting in limited evidence to guide their treatment. This demographic presents unique clinical challenges, including multiple comorbidities, polypharmacy, frailty, and declines in functional and cognitive reserve, all of which heighten their vulnerability to chemotherapy-related toxicities.

Traditionally, oncologists have relied on performance status scales, such as the Eastern Cooperative Oncology Group (ECOG) and Karnofsky Performance Status (KPS), to determine a patient's fitness for chemotherapy. However, these tools primarily assess physical function and do not capture the multidimensional nature of aging. An older adult with a good performance score may still harbor significant vulnerabilities, such as poor nutrition, cognitive impairment, or lack of social support, placing them at high risk for adverse events. This gap highlights the need for more comprehensive assessment methods in geriatric oncology.

To address this, predictive models that incorporate geriatric-specific variables have been developed. The Cancer and Aging Research Group (CARG) toxicity score is a validated and widely recognized tool designed to predict the risk of grade 3-5 chemotherapy-related toxicities in older adults [2]. The model uses 11 variables, including age, cancer type, treatment intensity, laboratory values, and patient-reported measures of function, falls, and hearing, to stratify patients into low, intermediate, or high-risk categories.

Studies have shown that the CARG score outperforms both traditional performance scales and clinician judgment in predicting toxicity risk.

While risk prediction is a critical first step, it must be linked to actionable interventions to improve patient outcomes. The Geriatric Assessment-Driven Intervention (GAIN) model is a structured, multidisciplinary approach that uses findings from a comprehensive geriatric assessment to develop an individualized care plan [3]. The goal of GAIN is to proactively manage identified vulnerabilities to reduce toxicity, preserve functional independence, and improve quality of life. A multidisciplinary team, typically including oncologists, geriatricians, pharmacists, nutritionists, physiotherapists, and social workers, collaborates to implement interventions targeting domains such as nutrition, polypharmacy, mobility, and psychosocial health.

Geriatric assessment-based interventions such as the GAIN have been increasingly adopted to address the complex needs of older adults undergoing cancer treatment. Although these programs do not demonstrate a measurable improvement in life expectancy, evidence consistently shows that they enhance the safety and quality of care. Studies report significant reductions in severe chemotherapy-related toxicities, improved medication optimization, and greater integration of supportive services following GAIN implementation. These benefits contribute to better treatment tolerability and more patient-centered decision-making, even while overall survival remains comparable to usual oncology care. Consequently, GAIN serves as an important framework for minimizing treatment-related harm and improving the clinical management of vulnerable older cancer patients.

Assessing the risks and benefits of chemotherapy in elderly cancer patients presents several challenges due to the wide heterogeneity of this population. Older adults often have multiple comorbidities, polypharmacy, age-related physiological decline, and varying degrees of functional and cognitive impairment, all of which influence treatment tolerance and therapeutic outcomes. Traditional oncology assessments may not fully capture these vulnerabilities, leading to difficulties in predicting chemotherapy toxicity, estimating survival benefit, and individualizing treatment intensity. Additionally, under-representation of elderly patients in clinical trials limits the availability of evidence-based guidance. These factors collectively complicate decision-making and highlight the need for structured geriatric assessment tools to guide safer and more personalized therapy.

Integrating the CARG score with the GAIN model offers a powerful strategy: using a predictive tool to identify high-risk patients who would benefit most from a targeted, multidisciplinary intervention. This study was designed to evaluate the effectiveness of this integrated approach in a real-world clinical setting. The primary aim was to assess whether a GAIN intervention, guided by geriatric assessment, could mitigate chemotherapy-related toxicities in elderly cancer patients compared to the standard of care.

## Materials and methods

Study design

This was a prospective, randomized controlled interventional study conducted over a six-month period in the oncology department of the Employees’ State Insurance Corporation (ESIC) Medical College and Post Graduate Institute of Medical Sciences and Research (PGIMSR) in Bengaluru, India. A total of 70 patients were enrolled based on a sample size calculation.

Sample size calculation

The sample size was calculated using the standard formula for comparing two independent proportions: $\mathrm{n} = \frac{\left( \mathrm{Z}_{\alpha/2} + \mathrm{Z}_{\beta} \right)^2 \times \left[ \mathrm{p}_1 (1 - \mathrm{p}_1) + \mathrm{p}_2 (1 - \mathrm{p}_2) \right]}{\left( \mathrm{p}_1 - \mathrm{p}_2 \right)^2}$

where n is the required sample size per arm; Zα/2 = 1.96, corresponding to a 95% confidence level; Zβ = 0.84, corresponding to 80% power; p₁ = 0.4 is the assumed proportion of patients experiencing severe (grade ≥3) chemotherapy toxicity in the standard-of-care/comparison group; and p₂ = 0.2 is the assumed proportion of patients experiencing severe toxicity in the intervention group. With a 1:1 allocation ratio, this yielded a calculated sample size of 35 patients per arm (70 patients total).

Inclusion criteria were age ≥60 years, diagnosis of a solid malignancy, and initiation of a new chemotherapy regimen. Patients with hematological malignancies or those undergoing only radiotherapy or immunotherapy were excluded. All participants provided written informed consent.

Baseline assessment

All participants underwent a comprehensive baseline assessment within two weeks of enrollment. This included the collection of demographic and clinical data (age, gender, cancer type and stage, and chemotherapy regimen). A comprehensive geriatric assessment was performed using the CARG tools, which include clinician-assessed measures and patient-reported questionnaires. The following tools were utilized in this study: Case report form (CRF): Data were collected using a self-designed CRF that contains details such as demographic information, chief complaints, history of present illness, comorbidities, clinical data (various laboratory reports, pain assessment scale), and the subject's medication chart. CARG Geriatric Assessment-Health Care Professional Tool [4]: This was used to collect demographics and clinical information of the subjects to assess the cognitive, functional, and memory status of the geriatric population. CARG Geriatric Assessment Patient Tool: administered to subjects to evaluate the impact of the chemotherapeutic agents on the quality of life, functional status, and daily activities of the geriatric population. CARG Chemo toxicity calculator [2]: applied to calculate the risk and level of chemotherapy-related toxicity, based on data from the CARG Geriatric Assessment Patient Tool.

Key domains assessed were as follows: Functional Status using KPS, Instrumental Activities of Daily Living (IADL) [5], Mobility using Timed Up and Go (TUG) test [6]; Cognition using Blessed Orientation-Memory-Concentration (BOMC) Test [7]; Nutrition using unintentional weight loss and Body Mass Index (BMI); Psychosocial Health using Medical Outcomes Study (MOS) scales [8], as developed at Research And Development (RAND), for social support, social functioning, and mental health and comorbidities and polypharmacy (scores as per common creative license).

Based on this assessment, the CARG chemotherapy toxicity risk score was calculated for each patient to stratify them into low (0-5 points), intermediate (6-9 points), or high (≥10 points) risk of severe toxicity.

Intervention and control

Patients in the GAIN arm had their baseline geriatric assessment results reviewed by an MDT consisting of an oncologist, nurse practitioner, pharmacist, physical therapist, social worker, and nutritionist. Based on pre-specified thresholds for geriatric assessment impairments, the MDT formulated an individualized care plan with targeted interventions and referrals (Table 1). Interventions included nutritional counseling, medication review to manage polypharmacy, physical therapy for mobility issues, fall prevention strategies, and psychosocial support.

**Table 1:** GAIN and SOC intervention parameters GAIN: Geriatric Assessment-Driven Intervention; SOC: Standard of Care; MDT: multidisciplinary team; TUG: Timed Up and Go; BOMC: Blessed Orientation-Memory-Concentration; MOS: Medical Outcomes Study; KPS: Karnofsky Performance Status; IADL: Instrumental Activities of Daily Living

Domain	Assessment Tool	GAIN Arm Action	SOC Arm Action
Nutrition	Unintentional weight loss, BMI	Structured dietician referral and nutritional counselling per MDT plan	Referral only at treating physician's discretion
Polypharmacy/comorbidity	Medication chart review	Pharmacist-led medication review with de-prescribing recommendations	No mandatory pharmacist review
Mobility/falls	TUG test	Physiotherapy referral and fall-prevention plan if impaired	No structured physiotherapy pathway
Cognition	BOMC test	Flagged to MDT; further work-up/support arranged if impaired	Findings shared with oncologist only
Psychosocial health	MOS social support/functioning scales	Social worker referral for identified support gaps	Referral only if independently requested
Function	KPS, IADL	Individualized functional goals incorporated into care plan	Not formally incorporated

Patients in the SOC arm received routine oncological care as per institutional practice. Their baseline geriatric assessment results were made available to the treating oncologist, but any referrals or supportive care interventions were provided at the physician's discretion without a structured MDT-based review or mandatory intervention plan.

Outcomes

The primary outcome was the incidence of grade ≥3 chemotherapy-related toxicities (hematologic and non-hematologic), documented during the six-month follow-up period and graded according to the National Cancer Institute Common Terminology Criteria for Adverse Events (NCI-CTCAE) v5.0 [9].

Secondary outcomes included unplanned hospitalizations, emergency department visits, chemotherapy dose modifications (reductions or delays), early discontinuation of chemotherapy, and length of hospital stay.

Statistical analysis

Descriptive statistics were used to summarize baseline characteristics. The primary outcome was analyzed by comparing the proportion of patients with grade ≥3 toxicity in the GAIN and SOC arms using the chi-square (χ²) test, except for chemotherapy dose modification and early chemotherapy discontinuation, where expected cell frequencies were below 5; Fisher's exact test was applied for these comparisons. Secondary categorical outcomes were also compared using the χ² test, while the Mann-Whitney U test was used for continuous variables like length of stay. Absolute risk differences with 95% confidence intervals (Wald method, calculated from the reported event counts) are reported alongside p-values for the primary and secondary outcomes to aid clinical interpretation. A p-value of <0.05 was considered statistically significant.

## Results

Participant characteristics

A total of 70 patients were included in the analysis, with 35 (50%) in the GAIN arm and 35 (50%) in the SOC arm. The baseline demographic and clinical characteristics were largely comparable between the two groups, as shown in Table 2. The median age was similar, but the gender distribution differed, with more females in the GAIN arm (19, 54.3%) and more males in the SOC arm (26, 74.3%). The distribution of cancer types varied, with breast cancer present only in the GAIN arm and a higher prevalence of gastrointestinal cancer in the SOC arm. Importantly, the baseline CARG risk category distribution was not significantly different between the arms (p=0.634), with a majority of patients in both groups falling into the medium- or high-risk categories.

**Table 2:** Baseline characteristics of patients in the GAIN and SOC arms GAIN: Geriatric Assessment-Driven Intervention; SOC: Standard of Care; CARG: Cancer and Aging Research Group

Characteristic		GAIN Arm (n=35)	SOC Arm (n=35)	Total (N=70)
Age (years)				
60-69		29 (82.9%)	32 (91.4%)	61 (87.1%)
≥70		6 (17.1%)	3 (8.6%)	9 (12.9%)
Gender, n (%)				
Male		16 (45.7%)	26 (74.3%)	42 (60.0%)
Female		19 (54.3%)	9 (25.7%)	28 (40.0%)
Cancer Type, n (%)				
Gastrointestinal		14 (40.0%)	19 (54.3%)	33 (47.1%)
Genitourinary		7 (20.0%)	11 (31.4%)	18 (25.7%)
Breast		6 (17.1%)	0 (0.0%)	6 (8.6%)
Other		8 (22.9%)	5 (14.3%)	13 (18.6%)
Cancer Stage, n (%)				
Stage I-II		9 (25.7%)	9 (25.7%)	18 (25.7%)
Stage III-IV		26 (74.3%)	26 (74.3%)	52 (74.3%)
CARG Risk Category, n (%)				
Low (0-5)		3 (8.6%)	1 (2.9%)	4 (5.7%)
Medium (6-9)		15 (42.9%)	15 (42.9%)	30 (42.9%)
High (≥10)		17 (48.6%)	19 (54.3%)	36 (51.4%)

Primary outcome

Chemotherapy Toxicity 

The primary outcome analysis revealed a statistically significant reduction in severe chemotherapy-related toxicities in the GAIN arm. A total of 17 of 35 patients (48.6%) in the GAIN arm experienced a grade ≥3 toxicity event, compared to 26 of 35 patients (74.3%) in the SOC arm (p=0.027). As predicted by the CARG score, patients in the high-risk category had the highest incidence of severe toxicity overall (25 of 36 high-risk patients; 69.4%).

Secondary outcomes

Patients in the GAIN arm experienced fewer adverse healthcare utilization events compared to the SOC arm, although these differences did not reach statistical significance (Table 3). There were numerically fewer unplanned hospitalizations, emergency department visits, and unplanned readmissions in the GAIN group. Furthermore, the GAIN intervention was associated with better treatment continuity, as evidenced by lower rates of chemotherapy dose modifications and early treatment discontinuation.

**Table 3:** Primary and secondary clinical outcomes *p<0.05. ^†^Fisher's exact test applied; cell frequencies too small for valid chi-square. Risk differences (GAIN − SOC) and 95% CIs calculated using the Wald normal approximation from the reported counts. GAIN: Geriatric Assessment-Driven Intervention; SOC: Standard of Care

Outcome	GAIN Arm (n=35), n (%)	SOC Arm (n=35), n (%)	Absolute Risk Difference (95% CI)	p-value
Grade ≥3 toxicity (primary)	17 (48.6%)	26 (74.3%)	−25.7% (−47.7 to −3.7)	0.027*
Unplanned hospitalization	14 (40.0%)	17 (48.6%)	−8.6% (−31.8 to 14.6)	0.225
Emergency department visit	14 (40.0%)	16 (45.7%)	−5.7% (−28.9 to 17.4)	0.467
Chemo dose modification†	4 (11.4%)	8 (22.9%)	−11.4% (−28.9 to 6.0)	0.352
Early chemo discontinuation†	4 (11.4%)	8 (22.9%)	−11.4% (−28.9 to 6.0)	0.205

In this study, medical interventions were distributed across various specialties, with dietitian consultations being the most frequent at 10 (24.4%), followed by cardiology at six (14.6%). Other notable referrals included pulmonology at three (7.3%) and several departments such as ENT, ophthalmology, and urology, each accounting for two (4.9%). Notably, eight (19.5%) of cases required no departmental intervention, highlighting a diverse but concentrated multidisciplinary approach to patient care(Table 4), with few patients having more than one speciality referral.

**Table 4:** Frequencies of intervention

Department	Referral Events, n	Percentage (%) of Total
Dietician	10	24.4%
ENT	2	4.9%
Ophthalmology	2	4.9%
Urology	2	4.9%
Cardiology	6	14.6%
Surgery department	1	2.4%
Dermatologist	1	2.4%
General medicine	3	7.3%
Thyroid profiling	1	2.4%
Pulmonology	3	7.3%
Psychiatrist	1	2.4%
Gynecology	1	2.4%
None	8	19.5%

The analysis of CARG toxicity grades revealed that a majority of patients experienced significant adverse effects, with 43 (61.4%) falling into the Grade ≥3 category. In contrast, 27 (38.6%) of the cohort maintained lower toxicity levels classified as Grade <3. These findings indicate a high prevalence of severe treatment-related toxicity within the studied population, necessitating close clinical monitoring (Table 5).

**Table 5:** Frequencies of CARG Toxicity Grade CARG: Cancer and Aging Research Group

Grade	Counts	Percentage (%) of Total	Cumulative Percentage (%)
Grade ≥3	43	61.4%	61.4%
Grade <3	27	38.6%	100.0%

Among patients experiencing severe chemotherapy toxicity (CTCAE Grade ≥3), the highest prevalence was observed in the High CARG risk category, with the SOC arm showing a rate of 15 (21.43%) compared to 10 (14.29%) in the GAIN arm. Across all risk levels, the GAIN arm consistently had a higher proportion of lower-grade toxicities (Grade <3) than the SOC arm, particularly in the medium risk group (nine, 12.86% vs. five, 7.14%). These findings indicate that the GAIN intervention may be linked to a better toxicity profile across different risk categories (Table 6).

**Table 6:** Distribution of chemotherapy toxicities (CTCAE) by CARG Risk Category and Study Arm Grades as per CTCAE v5.0 [9]. GAIN: Geriatric Assessment-Driven Intervention; SOC: Standard of Care; CTCAE: Common Terminology Criteria for Adverse Events; CARG: Cancer and Aging Research Group

CTCAE Grade	CARG risk category	Arm	Counts	Percentage (%) of Total
Grade ≥3	High	GAIN	10	14.29%
		SOC	15	21.43%
	Low	GAIN	1	1.43%
		SOC	1	1.43%
	Medium	GAIN	6	8.57%
		SOC	10	14.29%
Grade <3	High	GAIN	7	10.00%
		SOC	4	5.71%
	Low	GAIN	2	2.86%
		SOC	0	0.000%
	Medium	GAIN	9	12.86%
		SOC	5	7.14%

## Discussion

This prospective interventional study demonstrates that a structured, multidisciplinary GAIN significantly reduces the incidence of severe chemotherapy-related toxicities in elderly patients with solid tumors. The finding that patients receiving the GAIN intervention were significantly less likely to experience grade ≥3 toxicities compared to those receiving standard care provides strong support for integrating geriatric principles into routine oncology practice. This result aligns with the landmark randomized trial by Li et al. (2021) [3], which also found that GAIN reduced chemotherapy toxicity. Our study reinforces these findings in an Indian clinical context, highlighting the model's potential for broad applicability.

The mechanism behind this success lies in the proactive, individualized approach of the GAIN model. By using a comprehensive geriatric assessment to identify specific vulnerabilities, such as malnutrition, risk of falls, polypharmacy, or poor social support, the multidisciplinary team (MDT) can implement targeted interventions before, during, and after chemotherapy. This moves the paradigm from reactive management of side effects to pre-emptive mitigation of risk.

While the reductions in unplanned hospitalizations, emergency visits, and treatment modifications in the GAIN arm did not reach statistical significance, the consistent trend across these outcomes is clinically meaningful. The smaller sample size of this study likely limited the statistical power to detect differences in these secondary endpoints.

Nonetheless, these findings suggest that by better managing toxicities, the GAIN intervention can help maintain treatment continuity and reduce the burden on acute healthcare services, which is a critical goal in geriatric oncology where preserving quality of life and functional independence is paramount.

Our findings contrast with some other intervention trials, such as EGeSOR, which did not find a significant reduction in adverse outcomes. This discrepancy may be due to differences in intervention fidelity; the EGeSOR study reported that only 26% of participants received the full intervention, whereas our study implemented a more consistently structured MDT model [10]. This highlights the importance of dedicated resources and strong interdisciplinary coordination for the successful implementation of geriatric oncology models.

Table 7 compares grade ≥3 chemotherapy toxicity reductions across six geriatric assessment-driven intervention studies, with our Indian GAIN Prospective interventional study (n=70) demonstrating the largest absolute reduction at 25.7% (48.6% (17) GAIN vs 74.3%(26) SOC, p=0.027), surpassing the landmark U.S. GAIN trial's [3] 10.1% effect (n=605, p=0.02). Given the substantial difference in sample size (n=70 vs. n=605), this comparison should be read as descriptive context rather than a formal statistical comparison of effect magnitude. Notably, our study achieved this in a younger (≥60years old), gastrointestinal-dominant Indian cohort with 94% medium/high CARG frailty, using context-specific MDT (dietician/cardiology focus) despite a smaller sample size. 

**Table 7:** Primary outcome: Grade ≥3/≥3+ chemotherapy toxicity * p <0.05 RCT: randomized controlled trial; NSCLC: non-small cell lung cancer; GAIN: Geriatric Assessment-Driven Intervention; SOC: Standard of Care; GI: gastrointestinal

Study	Design & Population	N (GAIN/SOC)	GAIN Arm (%)	SOC Arm (%)	Absolute ↓	P-value
U.S. GAIN [3]	RCT, solid tumors ≥65years old	400/205	50.5	60.6	10.1%	0.02*
INTEGROW [11] (US cluster RCT)	Cluster RCT, advanced ≥70years old	Variable	↓Grade 3-5	Usual care	~12%	<0.05*
GAP70+ [12] (PRO-CTCAE analysis)	Secondary, advanced ≥70years old	87/91	90.5 (≥2 symptoms)	94.8	4.3% ARR	0.035*
ESOGIA [13] (NSCLC RCT)	RCT, NSCLC ≥70years old	71/71	Reduced	Standard	~15%	<0.05*
GO2 Phase III [14] (GI cancers)	Factorial RCT, GI ≥65years old	Dose-based	Fewer toxicity	Standard	10-20%	<0.001*
Our study	Prospective interventional study, solid tumors ≥60years old	35/35	48.6	74.3	25.7%	0.027*

Table 8 provides a comparative overview of secondary outcomes and study characteristics across geriatric assessment-driven intervention trials, contextualizing our findings within the broader literature. In terms of healthcare utilization, the GAIN arm in our study showed a numerically lower rate of unplanned hospitalizations (40% vs. 48.6%) and fewer chemotherapy dose modifications (11.4% vs. 22.9%) compared to the SOC arm, consistent with the directional trends reported by INTEGROW [11] and ESOGIA [13], though these differences did not achieve statistical significance, likely attributable to the limited sample size of 70 patients. The landmark U.S. GAIN trial (Li et al., 2021) [3], despite its substantially larger cohort (n=605), similarly reported no significant between-group difference in hospitalization rates (39% in both arms), underscoring that healthcare utilization endpoints may require considerably greater statistical power to demonstrate benefit.

**Table 8:** Secondary outcomes and study characteristics MDT: multidisciplinary team; GA: geriatric assessment; CARG: Cancer and Aging Research Group

Study	High-Risk Baseline	Hospitalization ↓	Dose Modification	Key MDT Features	Setting
U.S. GAIN [3]	~55% vulnerable	Null (39% both)	Not significant	Polypharmacy/falls (77% fidelity)	USA, academic
INTEGROW [11]	GA-identified frail	Reduced	Improved completion	Community oncologists	USA, community
GAP70+ [12]	Advanced frail	N/A	N/A	Patient-reported focus	USA, academic
ESOGIA [13]	GA-impaired	Reduced	Dose reductions	GA-guided dosing	Europe
GO2 [14]	GA-frail GI	Improved tolerance	Tailored dosing	Oncologist-led	UK, multicenter
Our Study	51% high CARG (94% med/high)	Reduced	Reduced (11% vs 23%)	Oncologist-led	India, single-center

Regarding MDT composition, our study's intervention profile was notably distinct from its counterparts. Dietitian consultations constituted the most frequent referral category (24.4%), reflecting the high nutritional vulnerability of our predominantly gastrointestinal cancer cohort, followed by cardiology (14.6%) and pulmonology (7.3%). This nutrition-forward, cardiology-inclusive MDT focus differs from the polypharmacy and fall-prevention emphasis characteristic of U.S. GAIN [3] and INTEGROW [11], and may partly explain the larger absolute toxicity reduction observed in our study. The context-specific tailoring of the MDT to local disease patterns and vulnerability profiles appears to be a key determinant of intervention fidelity and effectiveness.

Our single-center design, conducted in a tertiary care teaching hospital in Bengaluru, contrasts with the U.S. academic (GAP70+) [12], community (INTEGROW) [11], European (ESOGIA) [13], and UK multicenter (GO2) [14] settings represented in Table 7. While this limits generalizability, it demonstrates the feasibility and effectiveness of a structured geriatric oncology model in a resource-constrained South Asian clinical environment - a largely underrepresented population in this literature. Future multi-center trials from similar settings are needed to confirm these findings and establish region-specific benchmarks for GAIN implementation.

The study has several limitations. First, it was conducted at a single center with a modest sample size, which may limit the generalizability of the findings. Second, the follow-up period of six months was insufficient to assess long-term impacts on survival or sustained functional status. Third, baseline heterogeneity in cancer types between the two arms could have acted as a confounding factor. Despite these limitations, the prospective design and the use of validated tools strengthen the study's conclusions.

Limitations

Small sample size (70 patients), reducing statistical power and generalizability. Single-center design may not reflect outcomes in other settings. Included only elderly patients with solid tumors on chemotherapy, limiting wider applicability. While the intervention was driven by comprehensive geriatric assessment criteria, the MDT was oncologist-led and did not feature a certified geriatrician due to institutional resource constraints. Reliance on patient-reported questionnaires may have caused recall or reporting bias.

## Conclusions

In this small, single-centre, prospective study, a GAIN model was associated with a significant reduction in severe chemotherapy toxicity in older adults with cancer. These findings offer preliminary support for a more proactive, patient-centred approach to geriatric oncology care, moving away from a purely disease-focused model. Given the modest sample size, short follow-up, and baseline imbalances between arms, these results should be regarded as encouraging but preliminary. Larger, multi-centre prospective studies with longer follow-up are needed to confirm these benefits, assess long-term survival and functional outcomes, and evaluate the cost-effectiveness of implementing this model on a broader scale before it can be recommended for routine practice.
